# Does Frequency of ST-Segment Elevation Myocardial Infarction Presentation Impact Quality of Care?

**DOI:** 10.7759/cureus.1879

**Published:** 2017-11-26

**Authors:** Alex N Mazurek, Paul R Atkinson, Jaroslav Hubacek, Mark McGraw, Sohrab Lutchmedial

**Affiliations:** 1 Family Medicine, Saint John Regional Hospital/Dalhousie University; 2 Emergency Medicine, Saint John Regional Hospital; 3 New Brunswick Heart Centre, Saint John Regional Hospital/Dalhousie University; 4 Student, Saint John Regional Hospital

**Keywords:** myocardial infarction, electrocardiogram, quality, emergency care

## Abstract

Objectives

The volume of ST-Segment Elevation Myocardial Infarctions (STEMIs) presenting to an emergency department (ED) has been shown to affect treatment quality measures and patient outcomes. Almost half of ST-elevation-myocardial-infarction (STEMI) patients in New Brunswick (NB) present directly to community hospitals. This study seeks to determine if the quality of care received by STEMI patients presenting to EDs in NB is related to the volume of STEMI presentations at that center.

Methods

This retrospective registry-based study used data from the STEMI database at the New Brunswick Heart Centre (NBHC), identifying 1196 cases of STEMI in NB, Canada, between December 2010 and April 2013. Patients were stratified into three groups based on the annual volume of STEMIs seen at the presenting center. Quality of care determinants, consisting of the percent of cases adhering to door-to-ECG (D2E), ECG-to-needle (E2N), and door-to-needle (D2N) time guidelines were then compared between groups.

Results

The mean age of the 1188 cases identified was 61.3 years, 73.8% were male, and 69.0% received thrombolysis. There was no difference in the rate of guideline adherence between the high, medium, and low-volume centers. The total rates of guideline adherence were 43.7%, 44.9%, and 47.5% for the D2E, E2N, and D2N times, respectively.

Conclusion

We did not identify any relationship between the rates of adherence with STEMI care guidelines and the volume of STEMI patients presenting to a center. Adherence rates were lower than in previously reported series from other regions. Further efforts should be undertaken to identify the causes of delayed STEMI diagnosis and treatment in our population and to implement system changes to improve standards of care.

## Introduction

Advances in ST-elevation myocardial infarction (STEMI) care have resulted in improved reperfusion times and, consequently, a reduction in overall mortality [1]. However, STEMIs are still a major contributor to the overall burden of cardiovascular disease, with contemporary 30-day and 1-year mortality of approximately 4%-9% and 7%-13%, respectively [2-3]. Modern clinical practice guidelines now generally recommend three possible reperfusion strategies for patients presenting within 12 hours of symptom onset, depending on local resources and expected treatment delay: 

1. Primary percutaneous coronary intervention (pPCI) if the expected delay from first medical contact (FMC) to device deployment is less than 120 minutes;

2. Fibrinolysis (unless contraindicated) within 30 minutes of hospital arrival (if logistically unable to achieve pPCI), followed by urgent transfer to a PCI-capable facility for rescue PCI in patients with clinical evidence of failed reperfusion; or

3. Fibrinolysis followed by transfer for routine cardiac catheterization and angioplasty, as indicated, if clinical reperfusion was achieved (pharmaco-invasive approach) [4-5]. 

Many health regions in Canada cannot meet the strict criteria for pPCI, as only 63.9% of the population over 40 years of age lives within 60 minutes of a PCI-capable facility [6]. Additionally, timely access to pPCI in New Brunswick, Newfoundland, and Nova Scotia, the Atlantic Canadian provinces, is limited to 15.8%, 32.8%, and 36.8% of the population, respectively [6]. This geographic challenge, intrinsic to the sparsely populated Canadian landscape, has generated interest in describing real-world pPCI and fibrinolysis outcomes to ensure the optimization of STEMI care regardless of the population density of a region. Rural hospitals unable to meet the criteria for pPCI rely on a pharmaco-invasive (PhI) approach to STEMI management [7-8].

The short-term safety of this approach has been demonstrated previously in a prospective cohort study by Larson et al., which indicated no difference in 30-day mortality, stroke, reinfarction, or major bleeding between a cohort of patients in rural Minnesota receiving early fibrinolysis followed by immediate transfer for PCI, to those receiving pPCI [7]. The recently published EARLY-MYO randomized control trial also supported this approach and indicated that an early PhI strategy using early fibrinolytic followed by PCI was comparable to pPCI in low-risk patients [8]. Based on the growing support for early fibrinolysis and the knowledge that fewer than 9% of inter-hospital transfers meet the guidelines for door-to-balloon (D2B) time [9], the American College of Cardiology (ACC)/American Heart Association (AHA) now strongly recommends that in STEMI, fibrinolysis be administered early, unless contraindicated, followed by transfer to a PCI-capable hospital if there is an anticipated delay of 120 minutes from FMC to performing pPCI [4].

The most important determinant of STEMI outcome is time to reperfusion [10-11]. It has been demonstrated that for each 10-minute delay in door-to-needle (D2N) and D2B time, 6-month STEMI-related mortality increases by 0.3% and 0.18%, respectively [12]. Therefore, contemporary guidelines recommend that systemic delays be minimized to reduce total ischaemic time; specifically, that door-to-electrocardiogram (D2E) time be less than 10 minutes, D2N time less than 30 minutes, D2B time less than 90 minutes, and that regional STEMI care systems should strive for 90% adherence with these guidelines [13]. Unfortunately, it has been extremely challenging for centers to meet the ACC/AHA’s guidelines in practice, as evidenced by the reported low adherence with D2E and D2N guidelines in urban settings of 41% and 26%-47% of the cases, respectively [14-16] and mean D2E and E2N times of 8 minutes and 27 minutes, respectively [17].

Since almost half of STEMI patients present initially to emergency departments (EDs) in community hospitals, often staffed by family physicians [18-19] rather than tertiary referral centers, it is critical to determine if patients presenting with STEMI to community hospitals have similar treatment and outcomes. Recent research in Ontario has indicated that STEMI patients presenting to a low-volume center had a higher risk of being assigned a low triage priority, which, in turn, could be associated with delayed adherence to STEMI care guidelines [20-21]. Since extrapolation to the patients in New Brunswick, Canada, is unclear, the current study aimed to present an overview of STEMI care in the province and to investigate whether there were differences in quality outcomes, such as D2E, D2N, and ECG-to-needle (E2N) times between the tertiary referral centers and smaller community hospitals in the province.

## Materials and methods

Study design 

This is a retrospective registry-based study with data drawn from the STEMI database of the New Brunswick Heart Centre (NBHC) at the Saint John Regional Hospital (SJRH), the tertiary cardiac referral center in Saint John, NB, Canada. Ethics approval was granted by the regional research ethics board. An independent investigator collected and analyzed relevant data from the database, including all patients referred to the NBHC with a diagnosis of STEMI between December 31, 2010, and April 30, 2013, regardless of treatment modality. The investigator was also blinded to the identity of individual hospitals. Hospitals were grouped together by volume into high (H, greater than 50 STEMIs seen per year), medium (M, 20-50 STEMIs per year), and low (L, fewer than 20 STEMIs seen per year) centers. Cases were excluded if the referral center was not documented in the NBHC database. Further, for D2E and D2N determination, cases were excluded from the analysis if ECG changes initially resolved and then recurred, the presenting ECG was nondiagnostic for STEMI and was not recorded, it was a non-cardiac presentation, or the initial time of presentation could not be determined. The primary endpoint of the study was to compare the percentage of cases adhering to ACC/AHA 2013 guidelines for the D2E (≤ 10 mins), D2N (≤ 30 mins), and E2N (≤ 20 mins) times between the three groups. Since analysis of D2N and E2N times requires the patient to have undergone fibrinolysis, patients who did not undergo fibrinolysis were excluded from that part of the study.

For the most part, D2E times could easily be calculated, but because emergency medical service (EMS) providers do not perform prehospital 12-lead ECGs (PHECG) in New Brunswick, it was determined that for patients presenting to the hospital by ambulance, the time of first medical contact had to be set as the time the patient was registered in hospital as opposed to the time EMS providers attended on scene. Missing data were assumed to be outside our target values and were handled by assigning a label of "not within guidelines" and including it in the analysis.

Practice setting

The New Brunswick Heart Centre at the Saint John Regional Hospital sees an annual STEMI volume of approximately 500 cases and is the only center in the province with the infrastructure required to deliver PCI. Therefore, STEMI cases identified and initially treated in the province’s 28 other hospitals and health centers are transferred to the NBHC for further workup and eventual angiography. Due to the widely distributed population within the province, it is estimated that only 15.8% of the population lives within 60 minutes of the NBHC.

Database description

The NBHC has been prospectively tracking the delivery of care outcomes for STEMI patients presenting to the SJRH or transferred to the NBHC from other facilities since December 31, 2010. Treatment of STEMIs in hospitals outside of the NBHC is by protocol-based fibrinolysis (unless contraindicated) prior to transport to the NBHC. Patients are required to have symptom duration of less than 12 hours and ST-segment elevation greater than 1 mm in at least two contiguous leads to be included in the database. Full dose, weight-adjusted tenecteplase 30-50 mg intravenous (IV) bolus is administered in addition to loading doses of aspirin 325 mg PO and clopidogrel 300 mg PO (75 mg PO if older than 75 years). Patients are then anticoagulated with enoxaparin (30 mg IV bolus followed by 1 mg/kg SC 15 minutes later) or heparin (60 units/kg IV bolus followed by 12 units/kg/hr infusion). Transport to the NBHC is then facilitated as soon as possible and angiography arranged within 24 hours for those who undergo successful fibrinolysis or urgently for those who did not reperfuse as per clinical criteria. Patients presenting directly to the SJRH received either pPCI or fibrinolysis, according to local guidelines.

The current database tracks date of birth, gender, time of first presentation, mode of presentation, time of diagnostic ECG, time of fibrinolysis, time of presentation to the NBHC, time of angiography/PCI, reason for PCI (fibrinolysis failure, facilitated PCI, or pPCI), time of reperfusion, infarct location, other treatments (e.g., surgical management), length of stay, discharge disposition, and will eventually be linked with Vital Statistics NB for mortality outcomes. All patients with STEMI referred to the NBHC are included in the database regardless of treatment.

Statistical analysis

Comparisons of population characteristics and primary endpoints between the three hospital sizes were performed using one-way analysis of variance (ANOVA) for continuous and normally distributed variables and the chi-squared test for categorical variables. Normality was determined using the Shapiro-Wilk test for normality of data, and Levene's test was used to confirm the homogeneity of variances. For comparison of non-normally distributed data, the nonparametric Kruskal-Wallis H test (one-way ANOVA) was used. Statistical significance was defined as a p-value of less than 0.05. Statistical analyses were conducted with IBM Statistical Package for the Social Sciences (SPSS) software version 17.0 for Windows. Numerical data were reported as mean ± standard deviation (SD) for normally distributed data and when data were non-normally distributed, the 90th percentile value was also reported.

## Results

**Figure 1:**
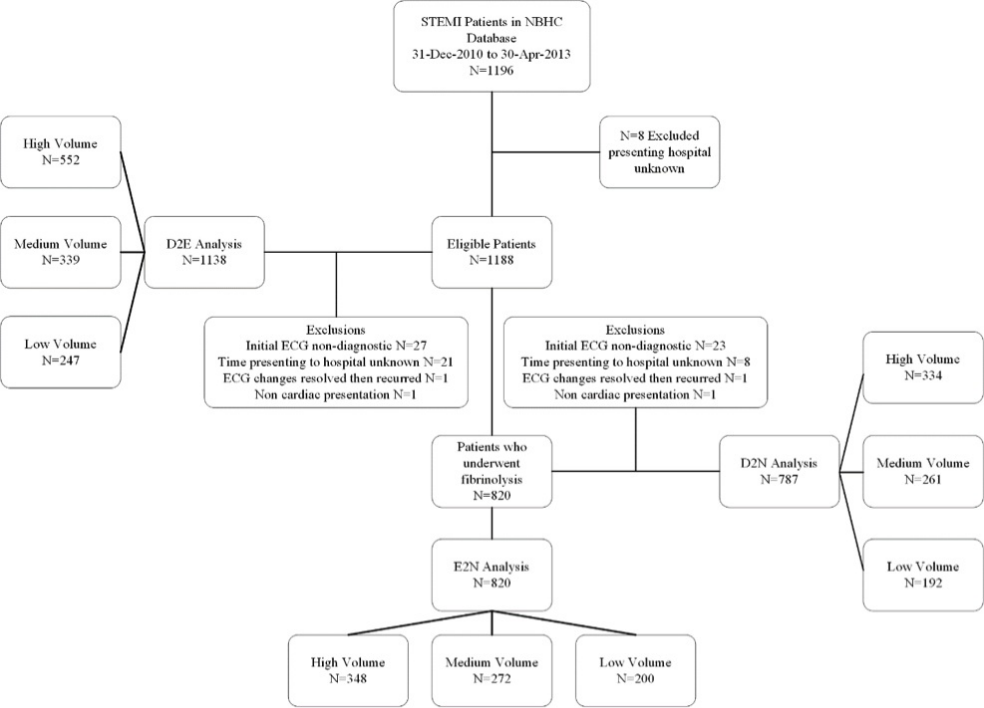
Study flow diagram showing the analysis of door-to-ECG (D2E); ECG-to-needle (E2N); and door-to-needle (D2N) times NBHC: New Brunswick Heart Centre; D2E: door-to-ECG; E2N: ECG-to-needle; D2N: door-to-needle; High Volume: greater than 50 STEMIs/year; Medium Volume: 20-50 STEMIs/year, Low Volume: less than 20 STEMIs/year

During the study period, 1196 patients with STEMI were recorded in the NBHC STEMI database. Eight of these patients were excluded, as their presenting hospital was unknown, resulting in 1188 eligible study patients. The mean age was 61.3±12 years and 875 patients were male (74%). The overall annual incidence of STEMIs presenting through the NBHC was 509. Province-wide, 820 (69%) patients underwent fibrinolysis during the study period and were then eligible for the E2N and D2N quality of care analyses. A total of 58, eight, and 41 cases were excluded from the D2E, E2N, and D2N analyses, respectively. Figure 1  provides a breakdown of patient allocation and exclusions.

The two most common reasons that cases were excluded was missing time of FMC and initial ECGs that were nondiagnostic and not recorded in the database. Presentation to the hospital was predominantly by walk-in (457 (38%)) or by ambulance (416 (35%)). For all eligible patients, the mean D2E time was 22.4±70.6 minutes, the 90th percentile D2E time was 32 minutes and was within guidelines in 497 cases (43.7%). The mean D2N time was 46.9±58.9 minutes, the 90th percentile D2N time was 85 minutes and was within guidelines in 374 (47.5%) of the time. Finally, the mean E2N time was 29.0±32.4 minutes, the 90th percentile E2N time was 57 minutes and was within guidelines in 368 (44.9%) of cases. Table 1 outlines key patient characteristics.

**Table 1:** Patient characteristics

Characteristics	All Patients N=1188	High Volume N=573 (48.2%)	Medium Volume N=357 (30.1%)	Low Volume N=258 (21.7%)	p-value
Number of centers, N (%)	28 (100)	3 (10.7)	5 (17.9)	20 (71.4)	<0.001
STEMIs/year, mean±sd	509±25.5	81.9±18.7	30.6±5.9	5.7±5.8	<0.001
Age, mean±sd	61.3±12.0	61.5±12.2	61.1±11.8	61.1±11.8	0.785
Male gender, N (%)	875 (73.8)	412 (72.0)	268 (75.3)	195 (75.9)	0.386
Fibrinolysis, N (%)	820 (69.0)	348 (60.7)	272 (76.2)	200 (77.5)	0.004
Presentation, N (%)					
Walk-in	457 (38.5)	207 (36.1)	137 (38.4)	113 (43.8)	0.238
Ambulance	416 (35.0)	237 (41.4)	99 (27.7)	80 (31.0)	0.002
Inpatient	31 (2.6)	16 (2.8)	13 (31.0)	2 (0.8)	0.067
Outpatient	2 (0.2)	1 (0.2)	0 (0)	1 (0.4)	1
Unknown	280 (23.6)	111 (19.4)	108 (30.3)	61 (23.6)	0.004
Other	2 (0.2)	1 (0.2)	0 (0)	1 (0.4)	1

Comparison of characteristics between center volume

Of the 1188 eligible patients, 573 (48.2%) from three hospitals were allocated to the H volume group, 357 (30.1%) from five hospitals to the M volume group, and 258 (21.7%) from 20 hospitals to the L volume group. The annual incidence of STEMI in each group was 81.9 (H), 30.6 (M), and 5.7 (L) units per year. The three groups had similar age, gender, proportion of walk-in, and inpatient presentation. The H volume group had a higher rate of presentation by ambulance than the M and L volume centers (41.4% vs. 37.7% & 31.0%, respectively, p = 0.002). 

Comparison of STEMI quality of care determinants between center volume

Door-to-ECG times were similar between the three groups (see Table 2). The mean D2E times were 20.1±46.0, 27.8±110.9, and 20.2±70.6 for the H, M, and L volume centers, respectively (p = 0.548). The 90th percentile D2E times were 29, 33, and 39 minutes for the centers, respectively, and all three groups had very similar rates of adherence to guidelines ranging between 42%-45% (p = 0.823).

**Table 2:** Comparison of door-to-ECG (D2E) times and adherence to guidelines by center volume

	All Patients (N=1138)	High Volume (N=552)	Medium Volume (N=339)	Low Volume (N=247)	p-value
D2E (mins), mean±sd	22.4±70.6	20.1±46.0	27.8±110.9	20.2±70.6	0.548
D2E (mins) 90^th^ %ile	32	29	33	39	-
D2E adherence N (%)	497 (43.7)	236 (42.8)	151 (44.5)	110 (44.5)	0.832

Fibrinolysis-specific quality of care determinants were also similar between the three groups, as outlined in Table 3 and Table 4, except for a small but significant difference in E2N times. 

**Table 3:** Comparison of ECG-to-needle (E2N) times and adherence to guidelines by center volume

	All Patients (N=820)	High Volume (N=348)	Medium Volume (N=272)	Low Volume (N=200)	p-value
E2N (mins), mean±sd	29.0±32.5	27.4±29.0	27.1±30.8	34.1±39.0	0.008
E2N (mins) 90^th^ %ile	57	49	51	64	-
E2N adherence N (%)	367 (44.8)	156 (44.8)	131 (48.2)	80 (40.0)	0.212

**Table 4:** Comparison of door-to-needle (D2N) times and adherence to guidelines by center volume

	All Patients (N=787)	High Volume (N=334)	Medium Volume (N=261)	Low Volume (N=192)	p-value
D2N (mins), mean±sd	46.9±58.9	46.5±61.2	45.8±60.8	49.2±51.9	0.523
D2N (mins) 90^th^ %ile	85	81	78	105	-
D2N adherence N (%)	374 (47.5)	168 (50.3)	125 (47.9)	81 (42.2)	0.198

Low volume centers had a mean E2N time of 34.1±39.0 minutes as compared to 27.1±30.8 for M, and 27.4±29.0 for H (p = 0.008). There was a similar difference in the 90th percentile E2N times of 49, 51, and 64 minutes for the H, M, and L groups, respectively. Despite this difference, there was no significant difference in the rate of adherence to E2N guidelines between the three groups with adherence rates ranging from 40%-48% (p = 0.212). There was also no significant difference in D2N times or adherence to guidelines. The mean D2N times were 46.5±61.2, 45.8±60.8, and 49.2±51.9 minutes for the H, M, and L volume groups (p = 0.523) and the 90th percentile times for the groups were 81, 78, and 105 minutes, respectively. Adherence to D2N guidelines ranged from 42.2% (L) to 50.3% (H) (p = 0.198). 

## Discussion

This study demonstrated no difference in the adherence to STEMI guidelines between the high-, medium-, and low-volume centers in New Brunswick. Adherence was fairly uniform across the three quality measures tested, ranging from 40%-50% and in line with estimates from published retrospective data that indicate 26%-47% adherence [14–16]. There was a significant difference in the mean E2N time across groups; however, this did not affect the E2N adherence to guidelines. Despite adherence data being in line with comparable retrospective publications, all three quality measures in this study (D2E 22.4, E2N 29.0, and D2N 46.9 minutes) were longer than currently published estimates from other centers where D2E, E2N, and D2N times ranged from 6.8 to 18.7, 18.7 to 27.0, and 29 to 46 minutes respectively [1,14,17,22].

The increased D2E time in this study may be influenced by the lack of initial nondiagnostic ECGs in the NBHC database. Since a small fraction of cases in the study had one or more nondiagnostic ECGs that went undocumented, a small amount of bias would have been introduced that might have artificially underestimated the rate of adherence to D2E time guidelines in our population. Given that previous studies have demonstrated that STEMI guideline concordance improves when the first ECG is diagnostic for a STEMI [15], it would have been helpful to have the initial ECG documented in the NBHC database. Prolonged D2E times are associated with increased D2N and D2B times and subsequent increases in 30-day mortality for patients presenting with nontraumatic STEMIs [16–18]. Studies concerning the improvement of D2E times have shown that low-cost measures focused on the initial point of contact for patient presentation can have a dramatic effect on STEMI quality of care. In one study, allowing ED registration staff to initiate the ECG request resulted in a 7-minute reduction in mean D2E time and increased D2E guideline adherence from 16% to 64% [23]. A similar study found that having a trained ECG technician greeting patients arriving at the ED and asking about their chief complaint resulted in a 20.8-minute reduction in D2E time [24]. Triage acuity has also been shown to affect D2E times for STEMI patients [20-21]. An Ontario study found that 25% of patients presenting with a STEMI were assigned a low triage priority, resulting in a 12.2-minute increase in D2E time and a 20.7-minute increase in D2N time [20-21]. Given the results of these studies, it is conceivable that a clinically significant reduction in D2E time for our study population could be achieved by implementing low-cost, frontline measures aimed at earlier ECG activation and recognition of patients at high risk for STEMI during triage.

The rate of fibrinolysis therapy in our study was 69.0%, substantially higher than the national average of 27.6%. This is not surprising given that only 15.8% of the population in New Brunswick over the age of 40 lives within 60 minutes of the NBHC [6]. Unfortunately, the mean D2N time in our study was 46.9 minutes, higher than other published estimates that ranged from 29 to 46 minutes [1,14,17,22]. D2N time has been shown to have a significant impact on both in-hospital and 30-day mortality for patients following STEMI, with in-hospital mortality increasing from 2.9% in patients with D2N times under 30 minutes to 4.1% with D2N times from 31 to 45 minutes, and 6.2% if D2N time exceeded 45 minutes [1,25]. Given the proportion of STEMI patients treated with fibrinolysis and the impact D2N time has on patient outcomes, reducing D2N time for our study population should be a priority. One factor likely to improve D2E and D2N times in our study population is to increase EMS utilization by patients with symptoms of ACS. Only 35.0% of STEMI patients in the current study arrived by EMS, far below the national average of 61.3% [26]. Studies have shown that presenting by ambulance cuts the time it takes to be seen by an ED physician in half, reduces the time from symptom onset to arrival at hospital by 31 minutes, and reduces D2E time by 3 minutes [26]. EMS-administered prehospital ECG (PHECG) for patients with STEMI symptoms has been shown to decrease D2N time by up to 36 minutes without substantially increasing time on scene, is a component of contemporary international guidelines for STEMI [4-5,27], and is being introduced in New Brunswick. These findings suggest that the implementation of a PHECG program, alongside increases in EMS utilization in the province, may result in reduced D2E and D2N times and improve STEMI outcomes. Increasing EMS utilization in the province would be challenging and require widespread public education through media campaigns; however, our results indicate that it represents a significant area for potential improvement in our province.

An important limitation of the current study is the inability to estimate accurately the total ischemic time from symptom onset. In our study, as well as many others on the topic, time from symptom onset is substituted for time of FMC, leaving approximately 60 minutes of prehospital ischemic time out of scrutiny [28]. Additional limitations of the current study include the omission of patients referred to out-of-province PCI centers, less than 3% of the overall volume, from the NBHC database, and the lack of initial non-diagnostic ECGs in patients who subsequently develop diagnostic ST changes. Furthermore, the treatment quality measures used in the current study are surrogates for the true mortality risk factor in STEMI, which is the total ischemic time from symptoms onset. 

## Conclusions

Measurements for the quality of care for STEMI did not vary by volume of STEMI patients seen. There was no difference in the rates of adherence to established STEMI care delivery guidelines between high-, medium-, and low-volume centers in the province of New Brunswick. However, the overall rates of adherence were well below the rates set by international guidelines. We recommend a further system analysis and changes at the levels of public awareness, triage, emergency department pathways, and communication and transfers within the provincial cardiac care system, to identify and correct factors that delay the diagnosis and treatment of STEMI patients across the province.
